# Using a smartphone app in the measurement of posture-related pupil center shift on centration during corneal refractive surgery

**DOI:** 10.3389/fcell.2023.1174122

**Published:** 2023-04-13

**Authors:** Wenbo Cheng, Li Li, Gang Luo, Yan Wang

**Affiliations:** ^1^ Department of Ophthalmology, The First Affiliated Hospital of Xinjiang Medical University, Urumqi, Xinjiang, China; ^2^ Department of Ophthalmology, Fujian Provincial Hospital South Branch, Fujian Provincial Hospital, Fuzhou, Fujian, China; ^3^ Schepens Eye Research Institute, Massachusetts Eye and Ear, Harvard Medical School, Boston, MA, United States; ^4^ Tianjin Eye Hospital, Tianjin Eye Institute, Tianjin Key Lab of Ophthalmology and Visual Science, Clinical College of Ophthalmology, Tianjin Medical University, Nankai University, Tianjin, China

**Keywords:** pupil, shift, centration, posture, cornea refractive surgery

## Abstract

**Purpose:** Pupil center is an important anchor point in corneal refractive surgery, which may affect by body position. This study investigated the feasibility of using a smartphone application in measurement of posture-related pupil center shifts.

**Methods:** Images of undilated eyes were captured for 25 participants (age: 18–38 years) at a distance of 40 cm in four body positions (seated, supine, right lateral, and left lateral) under controlled lighting conditions. During taking images, a smartphone application was used to guide positioning without head rotation and tilt. From the images, the location of the pupil center and pupil diameter with respect to the limbus boundary were measured.

**Results:** According to the data obtained by the smartphone application, pupil center was located slightly nasal and superior to the limbus center in the seated position, and it shifted more nasally and superiorly (*p* < 0.001, OD 0.54 ± 0.11 mm, OS 0.57 ± 0.14 mm) in the supine position. When body position switched between left and right lateral positions, the pupil centers of both eyes shifted along the direction of gravity (*p* < 0.05), and no significant shift occurred along the longitudinal axis. Moreover, pupil constriction was observed when the body position changed from seated to supine position (*p* < 0.001, OD 0.64 ± 0.57 mm, OS 0.63 ± 0.58 mm).

**Conclusion:** Posture-related pupil center shift may be larger than the error tolerance of centration in corneal refractive surgery, which might be difficult to measure by the existing instruments. An accessible application is necessary for evaluating the shift of pupil center and guiding centration during the surgery.

## Introduction

First postulated by Snellen in 1869, corneal refractive surgery has more recently become an alternative to dependence on contact lenses or spectacles for use in routine daily activities. Although there are several types of corneal refractive surgery, most involve laser vision correction (LVC), such as laser *in situ* keratomileusis (LASIK), laser epithelial keratomileusis (LASEK), femtosecond laser *in situ* keratomileusis (FS-LASIK), and small incision lenticule extraction (SMILE). Regardless of the treatment, centration remains a critical step during refractive procedures. Decentration of ablation may be associated with a significant increase in higher-order aberrations (HOAs) (Bueeler et al., 2003), decreases in quality of vision and contrast sensitivity, diplopia (Yap and Kowal, 2001), and night vision disturbances (Mrochen et al., 2001). Thus, proper centration of the treatment is essential during corneal refractive procedures.

The pupil center is widely utilized to determine centration during several corneal refractive procedures, such as LASIK and FS-LASIK. However, the location of the pupil center may shift. Yang (Yang et al., 2002) measured the location of the pupil center under photopic, mesopic and pupil dilation conditions, noting consistent temporal shifts in this location relative to the geometric corneal center as the pupil dilated. Another study (Mathur et al., 2014) reported that luminance and accommodation influence pupil size, although only the luminance change significantly affected the location of the pupil center. Moreover, some evidence suggests that changes in body position also induce pupil center shifts. Using wavefront measurements, Liu (Liu et al., 2013) observed such shifts between the seated-dilated state and the supine-undilated state during laser ablation, suggesting that the pupil center shifts temporally and superiorly. However, it was unable to determine whether changes in pupil diameter or body position induced a shift in the pupil center.

Before corneal refractive surgery, necessary examinations need be done in the seated position to check the pupil center location relative to the corneal center for prefect centration during the surgery. However, the surgery must be applied in the supine position due to the technical limitation, which may cause decentration by position-related pupil center shifts. Position-related pupil center shifts are hard to be measured by existing instruments, so this study turned to AI technology.

Therefore, a smartphone application is designed for investigating the pupil center shifts associated with 4 different body positions in this preliminary study. Although lateral body positions are not relevant to refractive surgery, the position were included to help postulate whether the pupil center shift may be related to gravity.

## Materials and methods

### Participants

The present study was conducted between July 2018 and May 2019, approved by the institutional review boards of each institution and conducted in accordance with the tenets of the Declaration of Helsinki. Informed consent was obtained from each participant following a detailed explanation of the nature and possible consequences of the study. Twenty-five individuals aged 18–38 years participated (Table 1). Inclusion criteria were as follows: 1) age ≥ 18 years, 2) clear corneas with no signs of opacity, and 3) absence of other ocular conditions. Exclusion criteria were as follows: 1) any corneal and iris anomalies, such as keratoconus or iritis, and 2) use of any ophthalmic or systemic medications.

**TABLE 1:** Subject characteristics.

Characteristics	All subjects included in this study (%) (*n* = 25)
Sex	
Female	10 (40.0)
Male	15 (60.0)
Ethnicity	
Caucasian	7 (28.0)
Asian	17 (68.0)
African American	1 (4.0)

### Measurement of pupil shifts in different body positions

**FIGURE 1:**
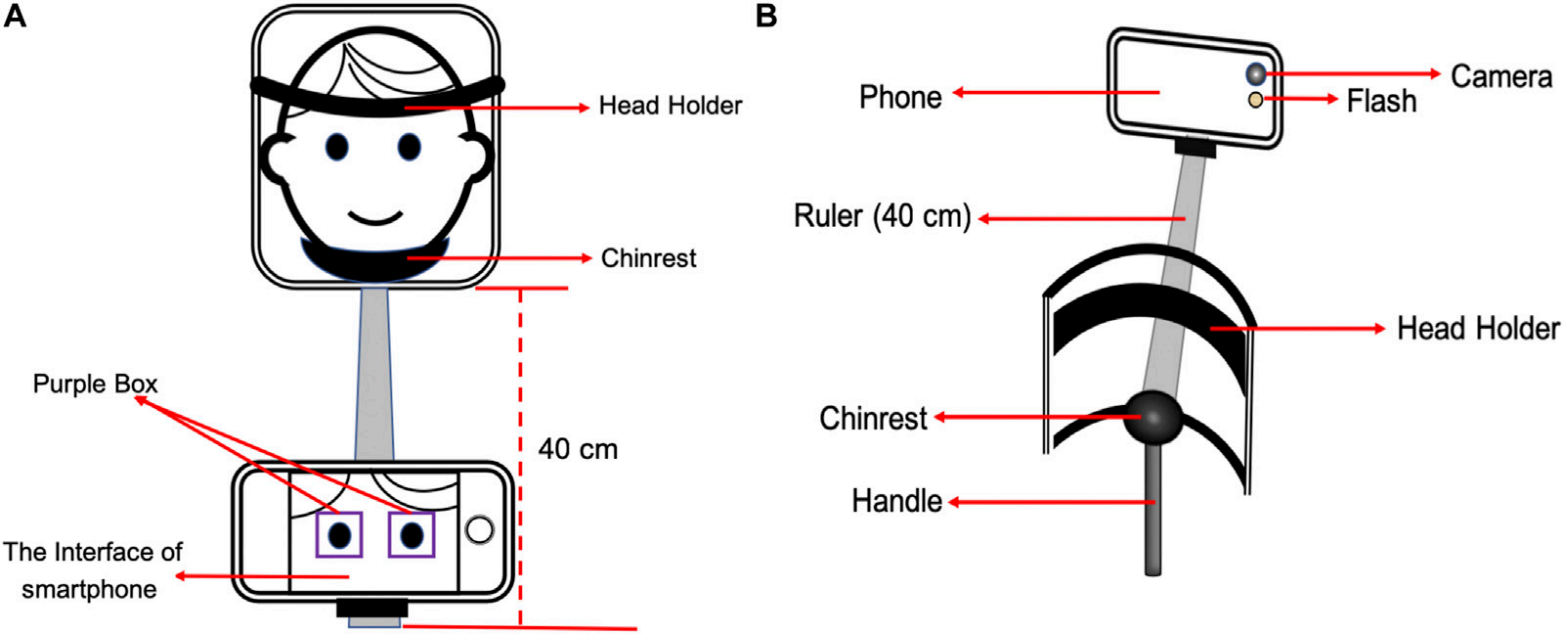
The device was made to fix the smartphone on the same location at 40 cm from the face. Subjects were asked to hold the device and focused on the camera flash during the whole process, putting the chin on the chinrest and sticking the forehead to the head holder **(A, B)**. A pair of purple boxes were plotted on the screen to help guide positioning without head rotation **(A)**.

Participants were asked to take four positions (seated, supine, right lateral, and left lateral), staring directly at the smartphone camera flash. A photo was captured including both eyes, using a smartphone application at a distance of 40 cm under the same illuminant condition. An apparatus was made to ensure that the distance and location were kept constant during image capture (Figure 1). Participants were instructed to hold the device, place their chin on the chinrest, and maintain contact between their forehead and the forehead bar during the experiment, even in lateral positions.

The smartphone application was designed for images taken to guide positioning and minimize head rotation and tilt. A pair of alignment boxes were plotted on the screen, the distance between them can be adjusted according to the pupil distance of each Participant (Figure 1). When taking images, eyes should be included in boxes with the eyelid margin parallel to horizontal borders. For good quality images, the limbus and pupil should be seen clearly, so that the boundaries can be marked without ambiguity.

### Image analysis

**FIGURE 2:**
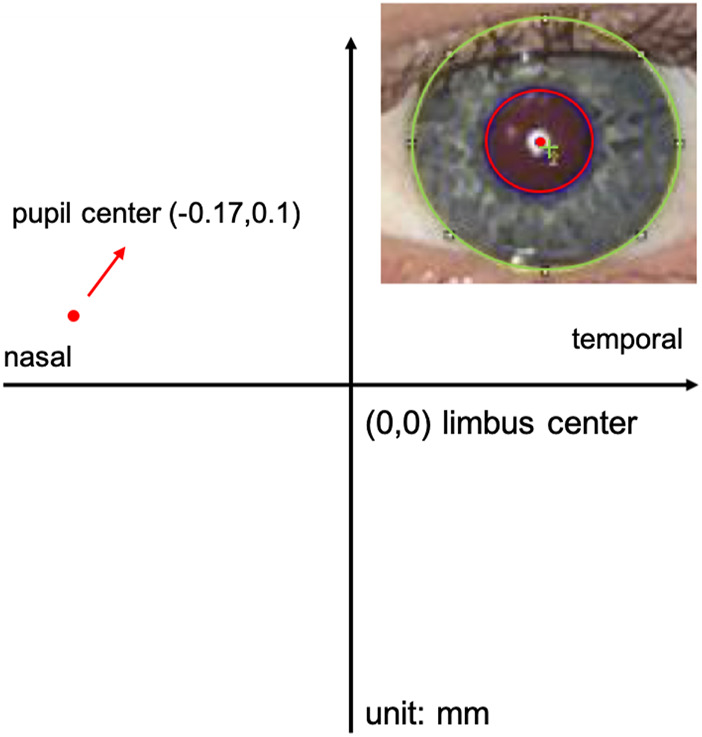
A good quality image is processed using Image J. The limbus and pupil of one eye can be accurately marked by Image J. Limbus center was considered as Origin (0, 0), and marked by green cross. Pupil center was marked by red dot.

Image J (Rawak Software, Inc., Germany) was used to mark the limbus and pupil rim, from which their centers were determined (Figure 2). Because that the limbus is a fixed biological landmark, the shifts in pupil center were calculated with respect to limbus center. We also measured pupil and limbus diameters in pixels, and then converted those measures to millimeters assuming the iris diameter is 11.8 mm for all eyes (Bergmanson and Martinez, 2017). The vector shift of the pupil center was calculated as the square root of the sum of the squares of the horizontal and vertical shifts.

### Data analysis

Analysis was conducted using SPSS (version 23.0; IBM Corp., Chicago, IL, United States). The results are expressed as the mean ± standard deviation (SD). The normality of all data distributions was confirmed using the Kolmogorov-Smirnov test (all *p* > 0.05) prior to further analyses. A two-tailed test was used to compare the magnitudes of the horizontal and vertical shifts of the pupil centers in all eyes. The paired-samples *t*-test was used to evaluate whether shifts were significantly different between right and left eyes. Repeated-measures one-way analyses of variance (ANOVA) with Bonferroni correction were used to evaluate differences between pupil diameters at each position. The level of statistical significance was set at *p* < 0.05.

## Results

### Pupil center location in the seated and supine positions

**FIGURE 3:**
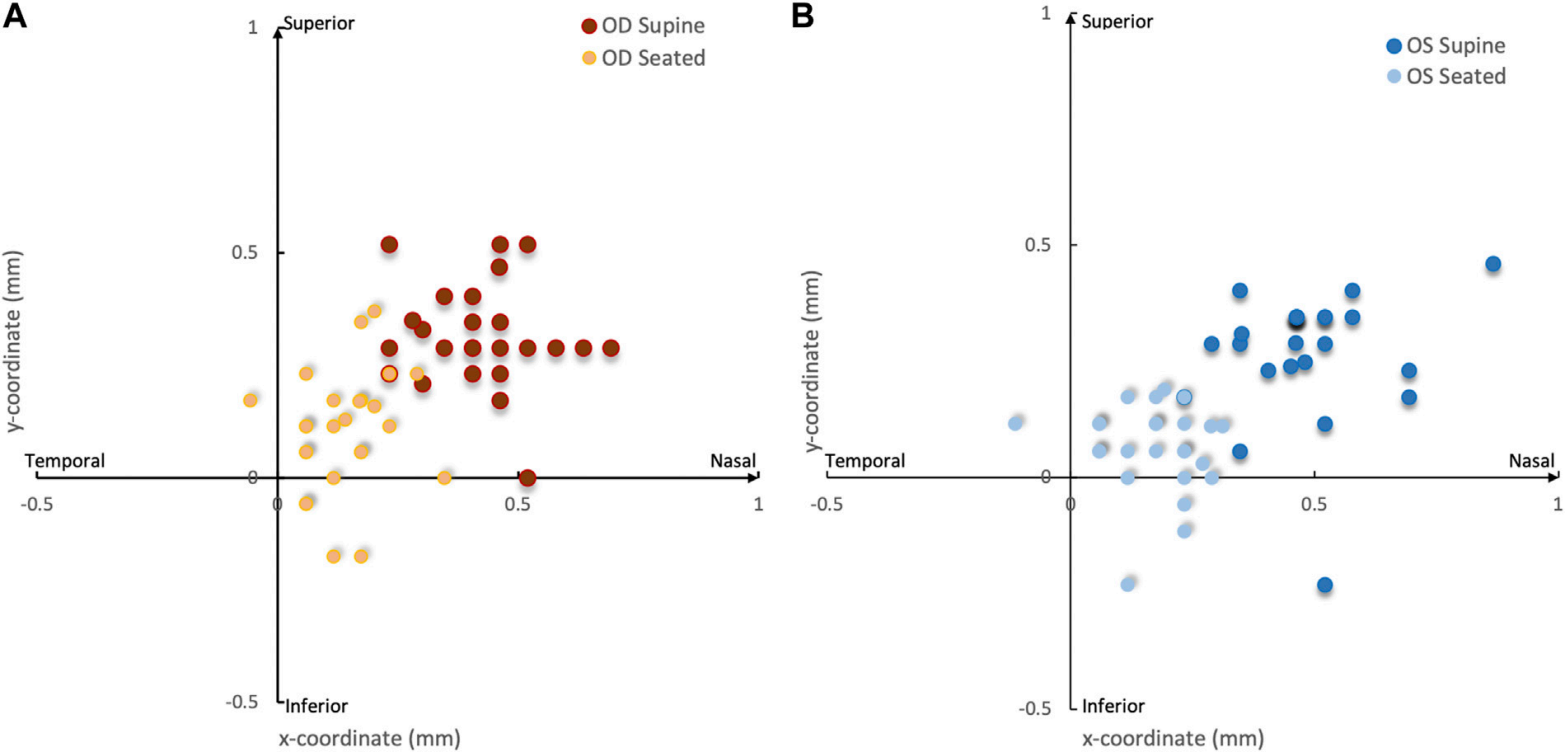
The pupil center location at seated and supine positions. **(A)** shows right eyes, and **(B)** shows left eyes. The coordinates (0, 0) represent the limbus center. The pupil center location of both eyes located slight superiorly and nasally, which shift more superiorly and nasally at supine position. Temporal, superior, inferior and nasal represent the direction of both eyes.

The location of the pupil center in the seated position is shown in Figure 3. They are slightly superior and nasal relative to the limbus centers. Considering the limbus center as the origin (0,0), the average location of the right pupil center was at (0.14 ± 0.09, 0.10 ± 0.13 mm), and the location of the left pupil center was at (−0.17 ± 0.10, 0.06 ± 0.10 mm) (Table 2). The off-center amplitude was almost the same for the two eyes (*p* = 0.997, 0.21 ± 0.10 mm for the right eye and 0.21 ± 0.07 mm for the left eye).

**TABLE 2:** The pupil center location at different positions.

Body position	Eye	X coordinate	Y coordinate	Amplitude
		Mean ± SD (mm)		
Seated	OD	0.14 ± 0.09	0.10 ± 0.13	0.21 ± 0.10
	OS	−0.17 ± 0.10	0.06 ± 0.10	0.21 ± 0.07
Supine	OD	0.42 ± 0.12	0.32 ± 0.12	0.54 ± 0.11
	OS	−0.48 ± 0.14	0.27 ± 0.14	0.57 ± 0.14
Supine-Seated	OD	0.28 ± 0.14 (*p* < 0.001)	0.21 ± 0.17(*p* < 0.001)	0.38 ± 0.16 (*p* < 0.001)
	OS	−0.31 ± 0.15(*p* < 0.001)	0.21 ± 0.15(*p* < 0.001)	0.40 ± 0.15 (*p* < 0.001)
Right lateral-seated	OD	−0.10 ± 0.14 (*p* = 0.011)	0.03 ± 0.15 (*p* = 0.373)	0.19 ± 0.13 (*p* < 0.001)
	OS	−0.26 ± 0.18 (*p* < 0.001)	0.02 ± 0.13 (*p* = 0.727)	0.31 ± 0.14 (*p* < 0.001)
Left lateral-seated	OD	0.21 ± 0.15 (*p* < 0.001)	0.06 ± 0.21 (*p* = 0.101)	0.28 ± 0.18 (*p* < 0.001)
	OS	0.13 ± 0.09 (*p* = 0.001)	0.04 ± 0.16 (*p* = 0.294)	0.21 ± 0.10 (*p* < 0.001)

OD, right eyes; OS, left eyes. Supine-seated position, shifts of pupil center from seated to supine position; right lateral-seated position, shifts of pupil center from seated to right lateral position; left lateral-seated position, shifts of pupil center from seated to left lateral position.

The pupil center was more nasally and superiorly when the position changed from seated to supine. On average, the right pupil center shifted to (0.42 ± 0.12, 0.32 ± 0.12 mm), and the left pupil center shifted to (−0.48 ± 0.14, 0.27 ± 0.14 mm). The off-center amplitude was almost the same for both eyes (*p* = 0.335, 0.54 ± 0.11 mm for the right eye and 0.57 ± 0.14 mm for the left eye).

In terms of the shift of each pupil, the right eyes shifted nasally by 0.28 ± 0.14 mm and superiorly by 0.21 ± 0.17 mm (*p* < 0.001), and the left eyes shifted similarly, nasally by −0.31 ± 0.15 mm and superiorly by 0.21 ± 0.15 mm (*p* < 0.001), when body position changed from seated to supine. Overall, the shift amplitude was about the same for both eyes (0.38 ± 0.16 mm for the right eye and 0.40 ± 0.15 mm for the left eye, *p* = 0.579).

### Pupil center shifts in the lateral position

**FIGURE 4:**
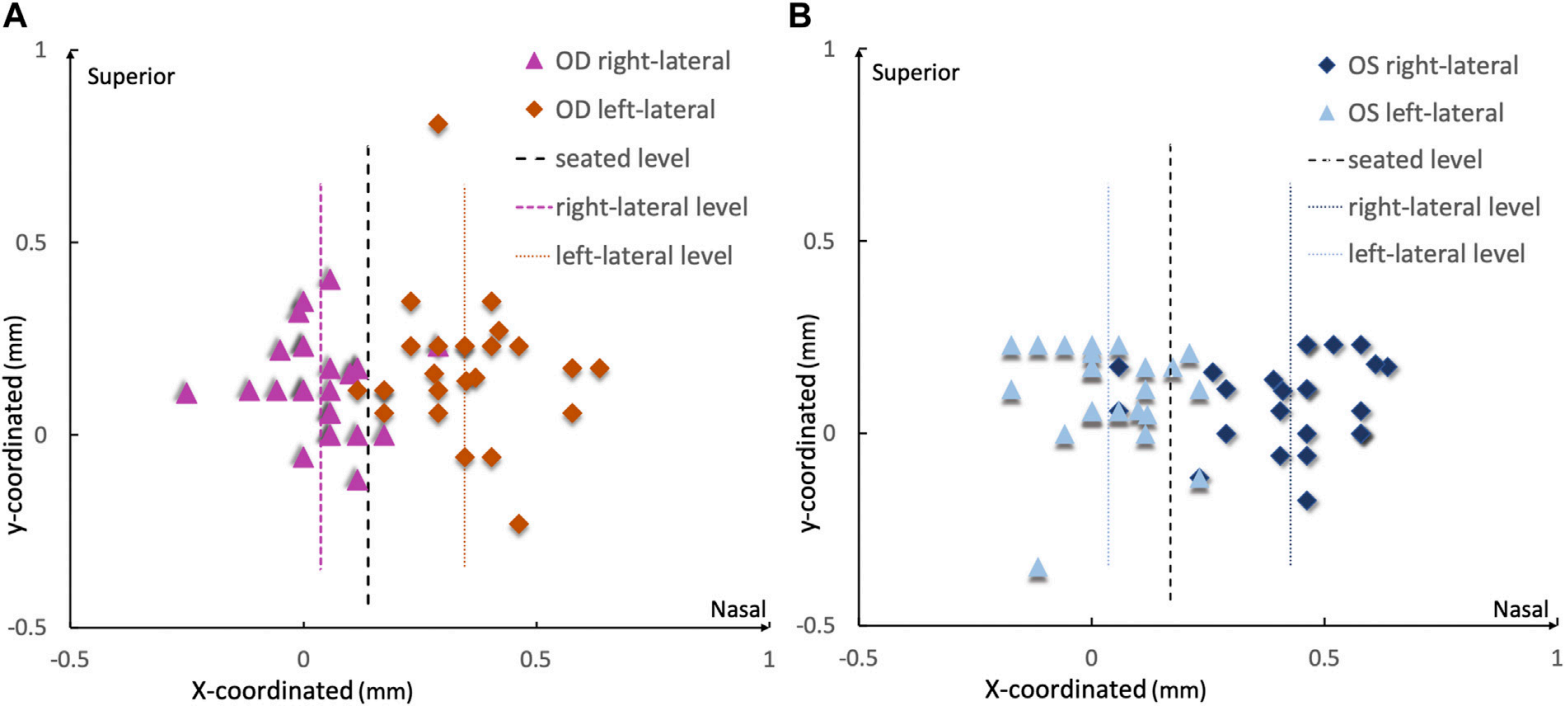
The pupil center shift at lateral position [**(A)** for right eyes and **(B)** for left eyes]. The coordinates (0, 0) represent the limbus center. When changed from seated to right lateral, pupil centers of both eyes shift to the right, which means nasal for left eyes and temporal for right eyes. Similarly, when switched from seated to left lateral, pupil centers of both eyes shift to the left, which means nasal for right eyes and temporal for left eyes. The dotted lines represent the average shifts at each position.

The results of pupil shift in lateral position are shown in Figure 4 and Table 2. When the body position changed from the seated to right lateral position, the X coordinates of both pupil centers shifted to the right side (0.10 ± 0.14 mm for the right eyes, *p* = 0.011, and 0.26 ± 0.18 mm for the left eyes, *p* < 0.001). Similarly, When the body position changed from the seated to left lateral position, the X coordinates of both pupil centers significantly shifted to the left side (0.21 ± 0.15 mm for right eyes, *p* < 0.001, and 0.13 ± 0.09 mm for the left eyes, *p* < 0.001). Interestingly, the nasal shift was larger than temporal shift for both left and right later positions (*p* < 0.001). In other words, the pupil in the upper position dropped down significantly more than the pupil in the lower position, when the bodies lied on one side. No significant shift was observed for the Y coordinates (*p* > 0.101).

### Changes in pupil diameter in different positions

**FIGURE 5:**
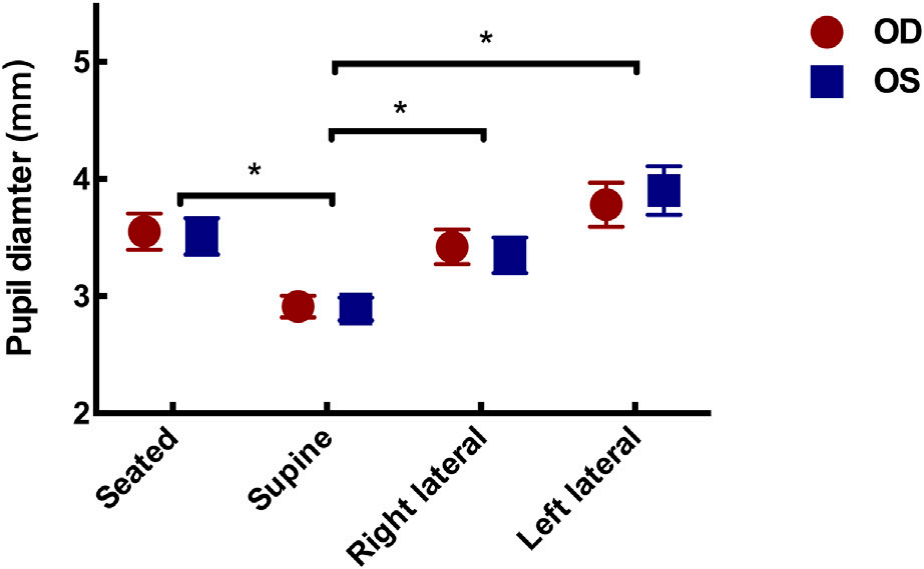
Pupil diameter change in different positions. The horizontal pupil diameter became constricting when the position changed from seated to supine (*p* = 0.09 for right eye, and 0.08 for left eye).

Interestingly, the pupil diameter in supine position was significantly than all the other positions (Figure 5). For instance, the pupil constricted by 0.64 ± 0.57 mm in the right eyes and by 0.63 ± 0.58 mm in the left eyes (*p* < 0.001) when the body position changed from seated to supine. There were no significant differences observed between the seated and right/left lateral positions (*p* > 0.05).

## Discussion

This study aimed to investigated the feasibility of using a smartphone application in measurement of posture-related pupil center location and pupil diameter. According to the data obtained by the application, the pupil center was located slightly nasally and superiorly with respect to the limbus center when participants were in the seated position (Liu et al., 2013). Interestingly, the pupil center shifted more nasally and superiorly when participants were in the supine position, which is the body position of patients in corneal refractive surgery. To investigate what might cause the shift, we also measured the pupil center location in lateral body positions. The pupil center shift due to different changes in the body position suggests that gravity might play a role—the pupils in both eyes shift down if one sits up (from supine position), shift to left in left lateral position, and shift right in right lateral position. It has been shown that gravity can influence vertical eye position and movements as a whole (Pierrot-Deseilligny, 2009), but we are not aware of any previous work showing gravity’s impact on sub-structures inside the eyes. It is even more mysterious that the gravity seemed to affect two eyes differently—it pulls the two pupils in temporal direction (i.e., in opposite direction) when one sits up in addition to pulling them in inferior direction, and it pulls the pupils nasally more than temporally in lateral body positions.

Moreover, it was also found that the pupil constricted when the body position changed from seated or lateral to supine. A previous study (Yang et al., 2002) showed that pupil size change may be associated with pupil center shift—when pupil diameter changed from 7.58 mm (dilated condition) to 4.06 mm (photopic condition), the pupil center shifted inferio-nasally by approximately 0.183 mm with respect to the limbus center. In our study, the pupil constriction due to body position change was much smaller than that in Yang’s study (Yang et al., 2002) only from 3.5 to 2.9 mm. However, but the pupil center shifted more (about 0.4 mm) and in superior-nasal direction rather than inferio-nasal direction (Figure 3). The difference suggests that the mechanism behind the shift may be different. In other words, the pupil center shift observed in this study was associated with body position, not due to pupil size change.

Our findings may have implications to corneal refractive surgery, in which centration of the treatment zone is key to optimizing visual outcomes, and the pupil center is always used to determine centration or as an important reference point. Since the location of the pupil center may be variable depending on body position, it is a question whether the centration in treatment should be based on upright or supine position. Previous studies (Porter et al., 2006) have reported that decentration during LASIK procedures increases the risk of HOAs postoperatively, which cannot be well explained by an inconsistency between preoperative aberration measurements over a dilated pupil and surgical correction over an undilated pupil. A study by Liu et al. (2015) found that the postoperative refractive outcomes would decrease if the deviation between corneal vertex and lenticule center was more than 0.3 mm. Because the deviation due to body position change may reach 0.4 mm, as we found in this study, we speculate that the pupil center shift induced by changes in body position may contribute to the increased risk of postoperative HOAs. Further studies are warranted to confirm if body position should be taken into consideration when determining the treatment center.

Interestingly, the pupil diameter became smaller in the supine position than in the other body positions under similar illumination conditions. Because the images were captured under the same photopic conditions with the room lights on and the phone camera fixed at the same location, changes in pupil diameter were unlikely to have been influenced by changes in illumination. Non-luminance-mediated (Joshi et al., 2016) changes in pupil diameter have been associated with some neuronal activities, possibly because the supine position evokes pupillary constriction in both eyes. However, there was no significant difference in pupil diameter in lateral positions. Further studies are required to determine the relationship between pupil diameter and body position.

## Limitations

The present study possesses some limitations of note. The pupil location and pupil diameter were normalized by each participant’s iris diameter, rather than measured directly. To help readers better understand the level of pupil shift, the iris diameter for all eyes was approximated to 12 mm, and the pupil shift was scaled linearly using the iris diameter as a reference. Such an approximation may cause 4% standard deviation in the pupil shift estimation due to the variability in iris diameter (11.8 ± 0.5 mm) (Hashemi et al., 2015). To improve accuracy, future studies should measure individual’s iris size if it is used as a reference. Another limitation is that the participants fell within a narrow age range (18–38 years). Therefore, we could not investigate the effect of age with a small sample in this preliminary study. Adolescents and older adults should be included in future studies.

## Conclusion

Our findings demonstrated that the smartphone application is feasible for evaluating the posture-related shift of pupil center and guiding centration during the surgery. According to the data, the pupil center shifted nasally and superiorly when the body position changed from seated to supine. Non-luminance-mediated pupillary constriction also occurred at the same time. Posture-related pupil center shift may be larger than the error tolerance of centration in corneal refractive surgery, which may affect postoperative visual quality following corneal refractive surgery or any treatment using the pupil center as a reference. Further studies need to confirm if the shifts in pupil center location that may occur with changes in body position should be taken into consideration.

## Data Availability

The raw data supporting the conclusion of this article will be made available by the authors, without undue reservation.
